# Inward rectifiers and their regulation by endogenous polyamines

**DOI:** 10.3389/fphys.2014.00325

**Published:** 2014-08-27

**Authors:** Victoria A. Baronas, Harley T. Kurata

**Affiliations:** Department of Anesthesiology, Pharmacology, and Therapeutics, University of British ColumbiaVancouver, BC, Canada

**Keywords:** inward rectifier, potassium channels, polyamines, voltage-dependent gating, ion channel block, channelopathy

## Abstract

Inwardly-rectifying potassium (Kir) channels contribute to maintenance of the resting membrane potential and regulation of electrical excitation in many cell types. Strongly rectifying Kir channels exhibit a very steep voltage dependence resulting in silencing of their activity at depolarized membrane voltages. The mechanism underlying this steep voltage dependence is blockade by endogenous polyamines. These small multifunctional, polyvalent metabolites enter the long Kir channel pore from the intracellular side, displacing multiple occupant ions as they migrate to a stable binding site in the transmembrane region of the channel. Numerous structure-function studies have revealed structural elements of Kir channels that determine their susceptibility to polyamine block, and enable the steep voltage dependence of this process. In addition, various channelopathies have been described that result from alteration of the polyamine sensitivity or activity of strongly rectifying channels. The primary focus of this article is to summarize current knowledge of the molecular mechanisms of polyamine block, and provide some perspective on lingering uncertainties related to this physiologically important mechanism of ion channel blockade. We also briefly review some of the important and well understood physiological roles of polyamine sensitive, strongly rectifying Kir channels, primarily of the Kir2 family.

## Overview

Inward rectification is a property of certain ion channels to preferentially conduct current in the inward direction (“into the cell”). Although many ion channel types exhibit some degree of rectification, this review will focus on the class of inwardly-rectifying potassium (Kir) channels that generate strongly rectifying potassium currents (i.e., with steep voltage dependence). In contrast to voltage gated potassium channels, which require membrane depolarization to open, strongly rectifying Kir channels remain active around the physiological resting membrane potential, and sharply diminish their activity upon membrane depolarization (Nichols and Lopatin, 1997; Lu, 2004; Hibino et al., 2010). This deviation from the outward current rectification observed for the classical “Hodgkin-Huxley” delayed rectifier potassium conductance led to the term “anomalous rectifier” in early literature describing ionic conductances (now known to be Kir channels) with strong inward rectification (Hutter and Noble, 1960). This unusual voltage dependence relative to most other ion channel types underlies the general functional role of strongly rectifying Kir channels, to contribute a significant potassium conductance when cells are not electrically excited and rapidly silence their activity in response to a depolarizing stimulus. In this way, Kir channels with strong rectification properties contribute to the maintenance of a resting membrane potential, but still allow cellular electrical excitation to proceed.

The mechanism of steeply voltage dependent inward rectification of Kir channels is now well understood to be blockade by endogenous intracellular polyamines (Ficker et al., 1994; Lopatin et al., 1994; Fakler et al., 1995). However, it is important to recognize that the Kir gene family comprises channels with diverse functional properties, and despite their name, many Kir channel types do not exhibit particularly strong or steeply voltage dependent polyamine sensitivity. We will focus this review on the mechanism and structural details of polyamine block of the Kir2 subfamily channels (which are particularly sensitive to polyamines), as well as some of the details of their physiological roles and disruption in genetic channelopathies. It is noteworthy that “weak” inward rectifiers (with shallow voltage dependence, and weak polyamine sensitivity) play many important physiological roles, impacting diverse processes such as hormone secretion (Koster et al., 2000; Choi et al., 2011), ion transport in the nephron (Simon et al., 1996), and control of ionic gradients in the inner ear (Scholl et al., 2009). Thus, we also refer readers to a recent broad review that provides an overview of the structure, function, and physiology of the entire Kir channel family as an excellent starting point for further discussion of other Kir channel types (Hibino et al., 2010).

## Discovery and characterization of polyamines as blockers of Kir channels

**Figure 1:**
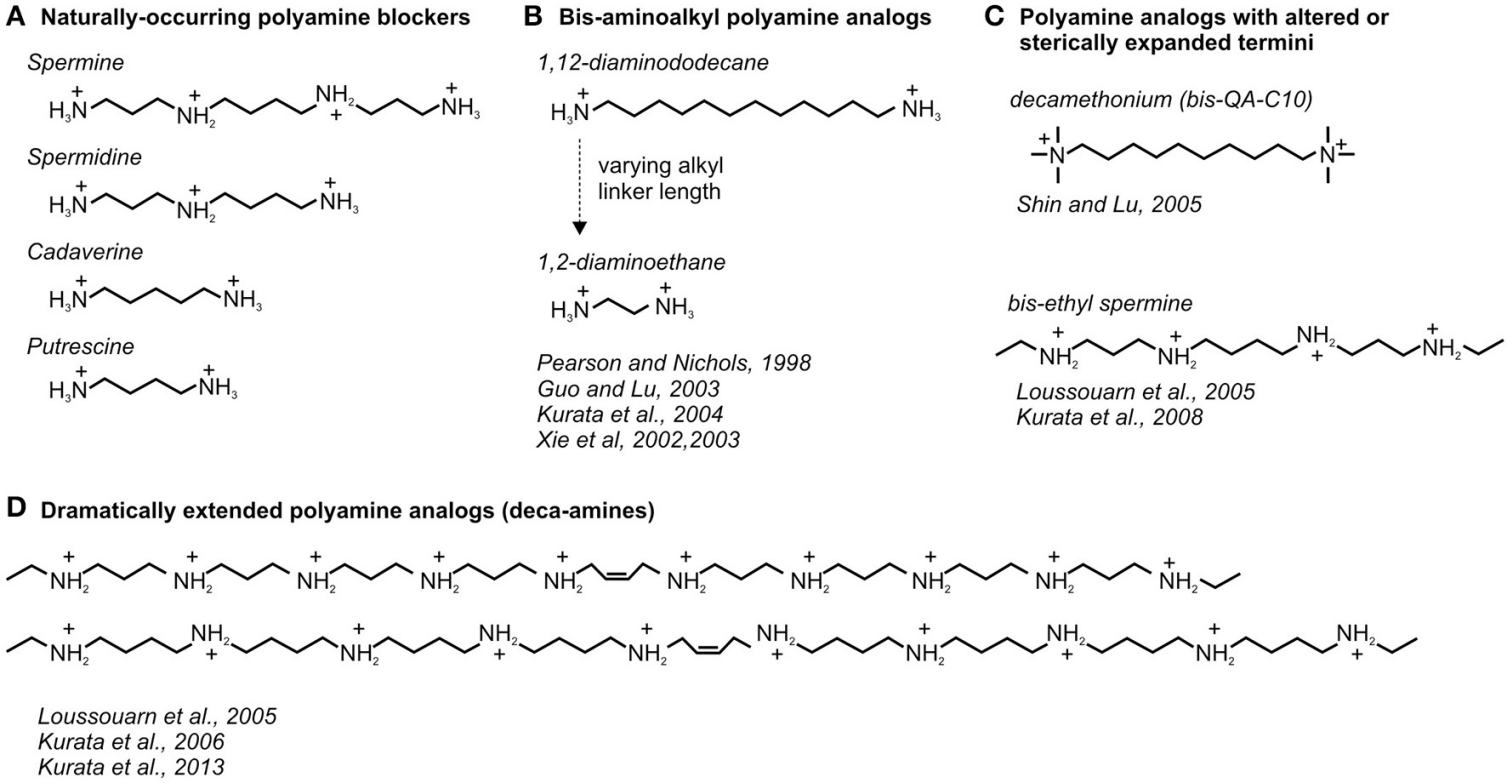
**Diversity of natural and synthetic polyamines used to study blockade of Kir channels**. Chemical structures of **(A)** naturally-occurring and **(B–D)** useful synthetic analogs of polyamine blockers of Kir channels are depicted with exemplar accompanying references.

The discovery of polyamine block as the mechanism of inward rectification was precipitated by the cloning of the first Kir channel genes (Ho et al., 1993; Kubo et al., 1993a,b), and the observation that inward rectification is largely an “extrinsic” property of Kir channels (Lopatin et al., 1994). By “extrinsic,” we mean that the strength of inward rectification can be reduced or even completely abolished when these channels are removed from the cellular environment (for example, by excision of membrane patches). Hints of the extrinsic nature of the process were apparent in early patch clamp studies of rectification of native strongly rectifying currents in cardiac myocytes, in which the strength of rectification was substantially diminished after excision of membrane patches (Matsuda et al., 1987; Vandenberg, 1987). However, the voltage dependent block produced by candidate mediators such as Mg^2+^ ions, did not match up with the steep voltage dependence of rectification observed in intact cells (Matsuda et al., 1987; Vandenberg, 1987). Fractionation of cell lysates, and their application to cloned Kir channels in excised membrane patches allowed for the identification of fractions that could restore inward rectification (Lopatin et al., 1994), eventually leading to the recognition of endogenously produced polyamines (and especially spermine, Figure 1A) as cellular elements that generate this electrical property (Ficker et al., 1994; Lopatin et al., 1994; Fakler et al., 1995). Among the naturally-occurring polyamines, spermine is the most potent Kir channel blocker and generates the steepest voltage dependence of block, followed closely by spermidine, while much shorter and less positively charged polyamines (cadaverine, putrescine) are less potent blockers and generate shallower voltage dependence (Section Blocker features essential for steep voltage dependent block) (Ficker et al., 1994; Lopatin et al., 1994; Fakler et al., 1995).

## Architecture of Kir channels

Availability of Kir channel clones, together with the recognition of polyamines as “gating particles” that underlie inward rectification, enabled a large body of work that has identified structural motifs of Kir channels that are essential for high affinity polyamine block (Nichols and Lopatin, 1997; Lu, 2004; Hibino et al., 2010). Although channel elements that contribute to polyamine block have been exhaustively studied, subtle but important details underlying this process continue to emerge. This section provides an overview of the three-dimensional structure of Kir channels, and the arrangement of residues that are essential for polyamine block.

### Transmembrane domain

**Figure 2:**
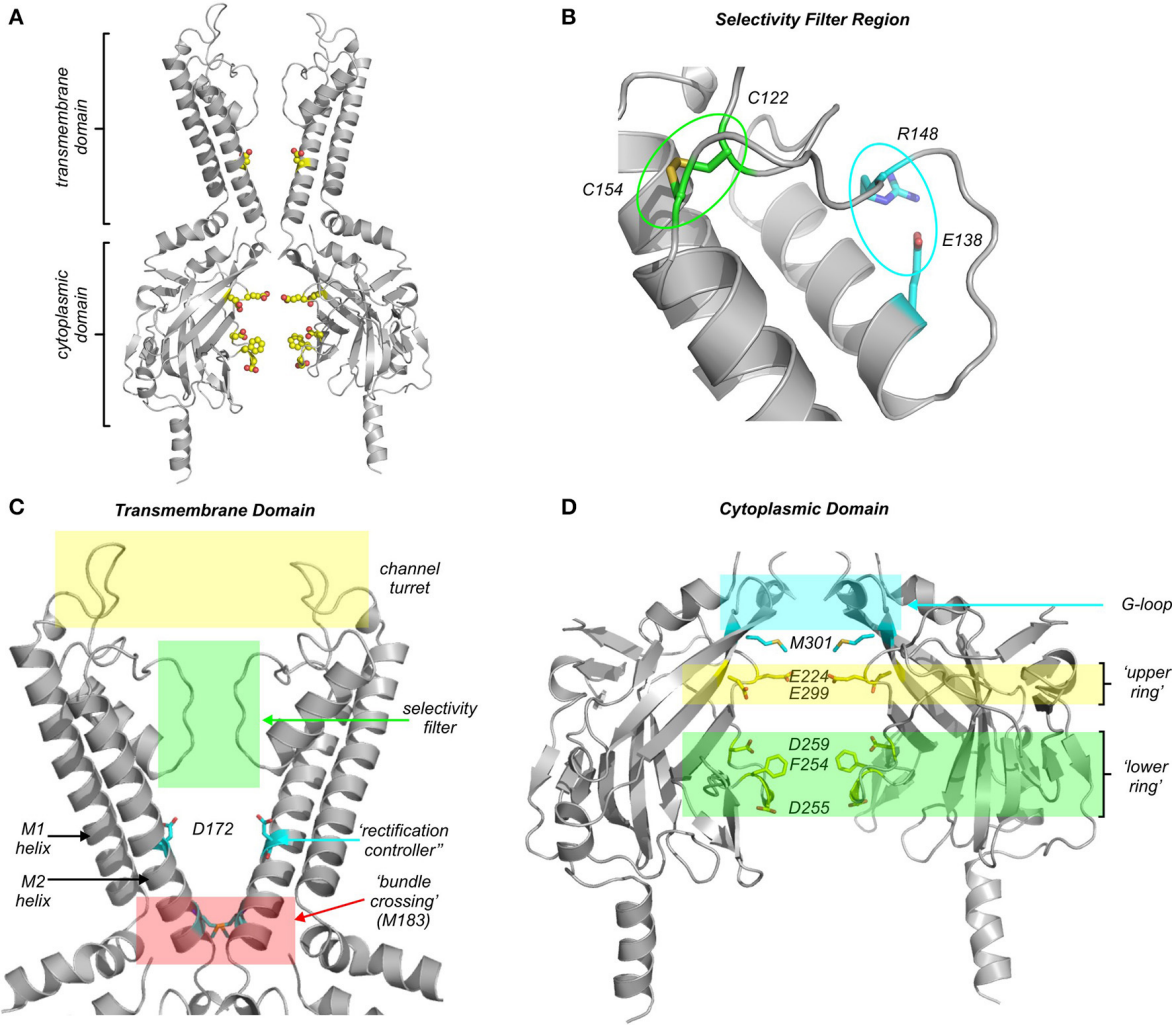
**Modular architecture of Kir channels, and the location of residues essential for polyamine block. (A)** Overall structural arrangement of the transmembrane (TMD) and cytoplasmic (CTD) domains of Kir channels. All structural models are constructed based on the Kir2.2 channel co-crystallized with di-C8-PIP2 (Hansen et al., 2011). Highlighted residues are D172, E224, F254, D255, D259, and E299 [labeled in more detail in **(C,D)**]. **(B)** Expanded view of the selectivity filter region, highlighting the disulfide bond (green) and salt bridge (cyan) that are conserved among Kir channels. **(C)** Expanded view of the transmembrane domain (TMD), with the “rectification controller” (Kir2.1 D172) residue, and bundle crossing constriction (M183) highlighted. **(D)** Expanded view of the cytoplasmic domain (CTD), where two rings of charge have been identified (lower ring: F254, D255, D259; upper ring: E224, E299, Kir2.1 numbering). Residue M301 is highlighted in cyan as a recently identified position that is mutated in a familial form of SQT3.

The earliest crystal structures of bacterial Kir (“KirBac”) channels revealed a modular architecture that is conserved between prokaryotic and eukaryotic Kir channels. These channels comprise a transmembrane domain (TMD) and a large cytoplasmic domain (CTD) that form an interface near the boundary between the cytoplasm and plasma membrane (Kuo et al., 2003; Nishida et al., 2007) (Figure 2A). The TMD is composed of two membrane spanning α-helices, an outer helix (M1) and a pore-lining inner helix (M2) (Tao et al., 2009; Hansen et al., 2011). These are connected by an extracellular turret region, a short pore helix and the selectivity filter, reminiscent of the transmembrane pore structure in crystal structures of other K^+^ channels such as KcsA and Kv1.2 (Figure 2B) (Doyle et al., 1998; Long et al., 2007). The sequence of the selectivity filter is similar between inward rectifiers and other potassium channel types, however there is some variability in the flanking sequences (Heginbotham et al., 1994; Nishida et al., 2007; Tao et al., 2009). Two unique features of the Kir channel selectivity filter region are an ion pair between the intracellular and extracellular sides of the selectivity filter (Figure 2B, cyan), and the presence of a conserved disulfide bond between the extracellular loops of the channel (Figure 2B, green). These are essential for the structural integrity of the selectivity filter and are required for normal channel function (Yang et al., 1997; Leyland et al., 1999; Cho et al., 2000). The turret in the outer pore restricts the size of the extracellular opening, and it has been proposed that this structural feature underlies the relative insensitivity of “classical” inwardly rectifying Kir2.x channels to toxins such as tertiapin (Figure 2C) (Jin and Lu, 1998; Hansen et al., 2011; Whorton and MacKinnon, 2011). Amino acids that contribute to the bundle crossing region, suspected to act as a gate at the base of the transmembrane helices (Figure 2C), vary between different Kir channel types. In some ligand-gated Kir subfamilies (i.e., Kir6.x, Kir3.x), a large aromatic amino acid (usually phenylalanine) is present and may be important for occluding the pore when channels are closed (Kuo et al., 2003; Sackin et al., 2005; Rojas et al., 2007; Khurana et al., 2011; Whorton and MacKinnon, 2011). In the prototypical strongly rectifying Kir2.x channels, the residue at the bundle crossing is a methionine (M183 in Kir2.1, Figure 2C) (Hansen et al., 2011).

### Cytoplasmic domain

N- and C-terminal segments of neighboring subunits interact to form a large cytoplasmic domain (CTD) that lines up beneath the TMD, extending the transmembrane pore to form a very long obligate permeation pathway for ions and blockers (Figure 2D). In the CTD, a conserved motif generating significant recent interest is the “G-loop” (Figure 2D) (Pegan et al., 2005). It is composed of small hydrophobic residues that may reduce the diameter of the pore in some channel states (Nishida et al., 2007), and certain mutations linked to Andersen-Tawil syndrome or familial short QT syndrome (both are Kir2.1-linked channelopathies, Section Cardiac arrhythmias related to Kir2.x channels and polyamine block) have been identified in the G-loop (Pegan et al., 2006; Hattori et al., 2012; Deo et al., 2013). Two glycines that flank the G-loop (G285 and G300 in Kir2.1) may confer a high degree of flexibility to this structure proposed to be involved in channel modulation by PIP2 and other ligands (Whorton and MacKinnon, 2011, 2013). Although the functional role of the G-loop remains incompletely understood, comparison of the growing variety of Kir channel crystal structures indicates structural diversity in terms of pore diameter at the level of the G-loop, raising the possibility that this motif undergoes relevant conformational changes during channel gating (Pegan et al., 2006; Nishida et al., 2007; Whorton and MacKinnon, 2011, 2013). In ligand-gated Kir channels, the CTD forms important binding sites for channel regulation by Gβγ (in Kir3.x “GIRK” channels) (Whorton and MacKinnon, 2013), or ATP (in Kir6.x “K_ATP_” channels) (Antcliff et al., 2005). Two features of the CTD are particularly important in the context of polyamine blockade. Firstly, the CTD contains numerous charged pore-lining residues that exert marked effects on the rate and stability of polyamine block (Section Regulation of polyamine block by residues in the cytoplasmic domain) (Shin et al., 2005; Fujiwara and Kubo, 2006; Kurata et al., 2007). Secondly, the CTD likely houses numerous occupant ions that are apparent in crystal structures of isolated domains and a chimeric Kir channel structure (Pegan et al., 2005; Nishida et al., 2007; Xu et al., 2009). This topic will be revisited (Sections Blocker features essential for steep voltage dependent block, Kinetic models of steeply voltage dependent polyamine block) when discussing processes that underlie steep voltage dependence of polyamine block.

### TMD-CTD interface

The CTD and TMD form a close interface generating a continuous permeation pathway that cannot be accessed through “fenestrations” in the sides of the channel (unlike the structurally dissociated cytoplasmic “T1” tetramerization domain and transmembrane domain of voltage-gated channels) (Long et al., 2007). The CTD-TMD interface is composed of contacts generated between multiple motifs, and is very sensitive to mutations, and so detailed functional roles of amino acids in the interfacial region have been difficult to distinguish using mutagenesis-based structure-function approaches (Li et al., 2013). In crystal structures of Kir2.2, binding of PIP2 appears to be an important contributor to formation of a stable CTD-TMD interface. In the absence of PIP2, Kir2.2 crystal structures exhibit a CTD that is disengaged from the TMD, whereas channels crystallized in the presence of PIP2 form a well-ordered TMD-CTD interface (Tao et al., 2009; Hansen et al., 2011). However, PIP2 has a much less obvious effect on crystal structures of Kir3.2 channels, which exhibit closely engaged CTD and TMD regions in both the presence and absence of PIP2 (Whorton and MacKinnon, 2011). Thus, although a role for PIP2 in TMD-CTD domain association is a possibility, we would be cautious to attribute the “disengaged” CTD and TMD apparent in Kir2.2 structures (without PIP2) to a specific physiological closed state (Hansen et al., 2011; Whorton and MacKinnon, 2011).

### Regulation of polyamine block by residues in the transmembrane domain

Structure-function studies employing mutagenesis have demonstrated that a single residue in the transmembrane region of the pore is particularly important for high-affinity, steeply voltage-dependent block by spermine and other polyamines. Specifically, Kir2.1 residue D172 (Figure 2C) was the first identified determinant of rectification properties, based on comparisons of a strong rectifier (IRK1, Kir2.1), with a weak rectifier (ROMK1, Kir1.1). In Kir2.1 this residue is an acidic aspartate (D172), whereas in Kir1.1 it is a neutral asparagine (N171). Swapping this residue between these functionally distinct channel types results in substantial transfer of their rectification properties (Lopatin et al., 1994; Lu and MacKinnon, 1994; Stanfield et al., 1994; Wible et al., 1994). Kir2.1[D172N] exhibits decreased affinity for spermine (although not as insensitive as Kir1.1). More convincingly, introduction of an acidic side chain in Kir1.1[N171D] converts it to a strongly rectifying channel with steeply voltage dependent polyamine sensitivity (Wible et al., 1994). The important influence of this residue has led to it being coined the “rectification controller” (Nichols and Lopatin, 1997). The specific location of the “rectification controller” has now been described in atomic resolution detail from the crystal structures of several inward rectifiers (Figure 2C), in the center of the inner cavity, between the selectivity filter and the bundle crossing region. An aspartate from each of the four subunits creates a ring of negative charges with which one or more amines from a polyamine blocker could potentially interact. However, the detailed features of spermine binding in the inner cavity have not yet been observed in a crystal structure of a strongly rectifying Kir channel.

Despite its strong influence on rectification properties, it is important to note that the “rectification controller” does not exert an “all-or-none” effect. Some Kir channels that lack an acidic residue at the “rectification controller” (e.g., Kir3.2) have been reported to exhibit fairly strong rectification properties, while the Kir2.1[D172N] mutation weakens (but does not abolish) polyamine binding (Wible et al., 1994; Yi et al., 2001; Guo et al., 2003). These observations indicate that other residues within the pore also make significant contributions to polyamine binding. Furthermore, it is noteworthy that introduction of polyamine sensitivity with an inner cavity acidic substitution is not very position sensitive. For example, introduction of an acidic side chain at *any* pore-lining position in the otherwise weakly rectifying Kir6.2 channel is able to strengthen spermine affinity and generate steeply voltage dependent block (Kurata et al., 2004). A possible implication of this is that the spermine binding site may not involve a highly defined architecture of interacting residues. Lastly, stabilization of spermine in its deepest blocked state is not solely dependent on acidic residues in the inner cavity. For instance, an alanine scan of the pore region of Kir2.1 identified functional contributions of Kir2.1 residues F174, I176, and M183 in steep inward rectification (see Section Divergent models of spermine binding in the inner cavity site), although each of these positions has a far smaller influence than the “rectification controller” D172 (Xu et al., 2009).

### Regulation of polyamine block by residues in the cytoplasmic domain

While the “rectification controller” is clearly an important determinant of polyamine block, chimeric studies also helped to identify residues in the cytoplasmic domain that are involved in polyamine block. Notably, interchanging the cytoplasmic domains of Kir2.1 and Kir1.1 resulted in partial transfer of rectification properties (Taglialatela et al., 1994). Further mutational analysis identified two residues in Kir2.1 (E224 and E299) as important determinants of rectification (Taglialatela et al., 1995; Yang et al., 1995; Kubo and Murata, 2001; Guo and Lu, 2003). Subsequent crystallographic studies illustrated that these two residues are located in the “upper” portion of the CTD, lying just below the G-loop, and close to the bundle crossing region of the TMD (Figure 2D). The carboxylate side chain of E225 in Kir2.2 (analogous to E224 in Kir2.1) creates a ring ~9 Å in diameter, with E300 (analogous to E299 in Kir2.1) occupying the space between adjacent E225 residues (Tao et al., 2009; Hansen et al., 2011). Neutralization of these glutamates (most commonly with the E224G and E299S mutations) markedly slows the kinetics of spermine block, and causes reduced spermine affinity (Taglialatela et al., 1995; Guo and Lu, 2003; Fujiwara and Kubo, 2006). However, interpretation of these effects is complex due to the presence of multiple distinct polyamine binding sites in Kir2.1 (see Section Kinetic models of steeply voltage dependent polyamine block) (Lopatin et al., 1995; Xie et al., 2003; Shin et al., 2005). An additional complication of interpretation arises because these mutations result in channels which exhibit intrinsic inward rectification and smaller single channel conductance (Kubo and Murata, 2001; Xie et al., 2002; Fujiwara and Kubo, 2006).

Crystallization of isolated cytoplasmic domains and full length eukaryotic Kir channels has led to further identification of pore-lining residues that impact spermine binding (Figure 2D). Two acidic pore-lining residues close to the cytoplasmic entrance of the CTD (D255 and D259) form a “lower ring” of charge that contributes to polyamine block (Figure 2D) (Pegan et al., 2005). Further analysis of D255 in this cluster demonstrated that it predominantly controls the kinetics of polyamine block, with little effect on overall affinity (Kurata et al., 2007). Mutation of a neighboring aromatic residue, F254 in Kir2.1, which constricts the pore to ~10 Å near the cytoplasmic entrance, produces very similar effects, primarily altering blocking kinetics but not affinity for spermine in the TMD binding site near D172 (Shin et al., 2005; Xu et al., 2009). Kir2.1 residue F254 has been described as a “gasket” that may minimize passage of K^+^ ions while polyamines occupy the cytoplasmic domain and thereby contribute to steep voltage-dependent block (Xu et al., 2009). However, a counterpoint to this idea is that the F254 “gasket” is not present in channels such as Kir6.2 and Kir1.1, although both can exhibit steeply voltage-dependent rectification after the introduction of an acidic residue in the rectification controller position (Wible et al., 1994; Shyng et al., 1997).

Overall, both the “upper ring” formed by E224/E299, and the “lower ring” formed by D255/D259/F254, exert significant control over the kinetics of spermine block. Of these, only E224 and E299 appear to significantly influence overall stability of spermine binding (mediated primarily by the “rectification controller” interaction in the TMD). This may be due to the closer proximity of this E224/E299 “upper ring” to the transmembrane domain. However, in the context of considering the functional contributions of different residues to polyamine block, it is noteworthy that many amino acids in the Kir pore can exert significant long range effects (Robertson et al., 2008). This was highlighted in a residue-by-residue decomposition of electrostatic contributions of each amino acid in Kir channel structures, and is particularly important to consider when using mutagenesis studies to attempt to define locations of blocker binding sites, as the impact of mutations of charged amino acids may not be restricted to their immediate vicinity (Robertson et al., 2008).

### Blocker features essential for steep voltage dependent block

In addition to characterization of channel residues involved in polyamine block, detailed characterization of a wide variety of polyamine analogs has led to a description of specific structural requirements of blockers for generation of steeply voltage dependent block, and allowed for diverse approaches to investigate the mechanistic basis of polyamine block (some of the more useful and informative analogs are illustrated in Figures 1B–D). The effects of polyamine analogs of different lengths and valence highlight that movement of charged polyamines through the transmembrane field cannot solely account for the steeply voltage dependent rectification of Kir2.x channels. For example, replacing two amines for hydrocarbons in tetravalent spermine yields a bivalent compound (1,12-diaminododecane, Figure 1B) that recapitulates the steep voltage dependence of spermine block (effective valence of ~4–5 elementary charges) (Pearson and Nichols, 1998; Guo et al., 2003; Kurata et al., 2004). In addition, bivalent (bis-amino) compounds with chains as short as 9 carbons can generate similarly steep voltage dependent block (Pearson and Nichols, 1998; Guo and Lu, 2003). Since this class of polyamines (“bis-amines”) carries only two charges (indicating a maximum effective valence of 2 if they move entirely through the transmembrane field), blocker migration across the membrane voltage gradient cannot account for the steep voltage dependence that is observed.

**Figure 3:**
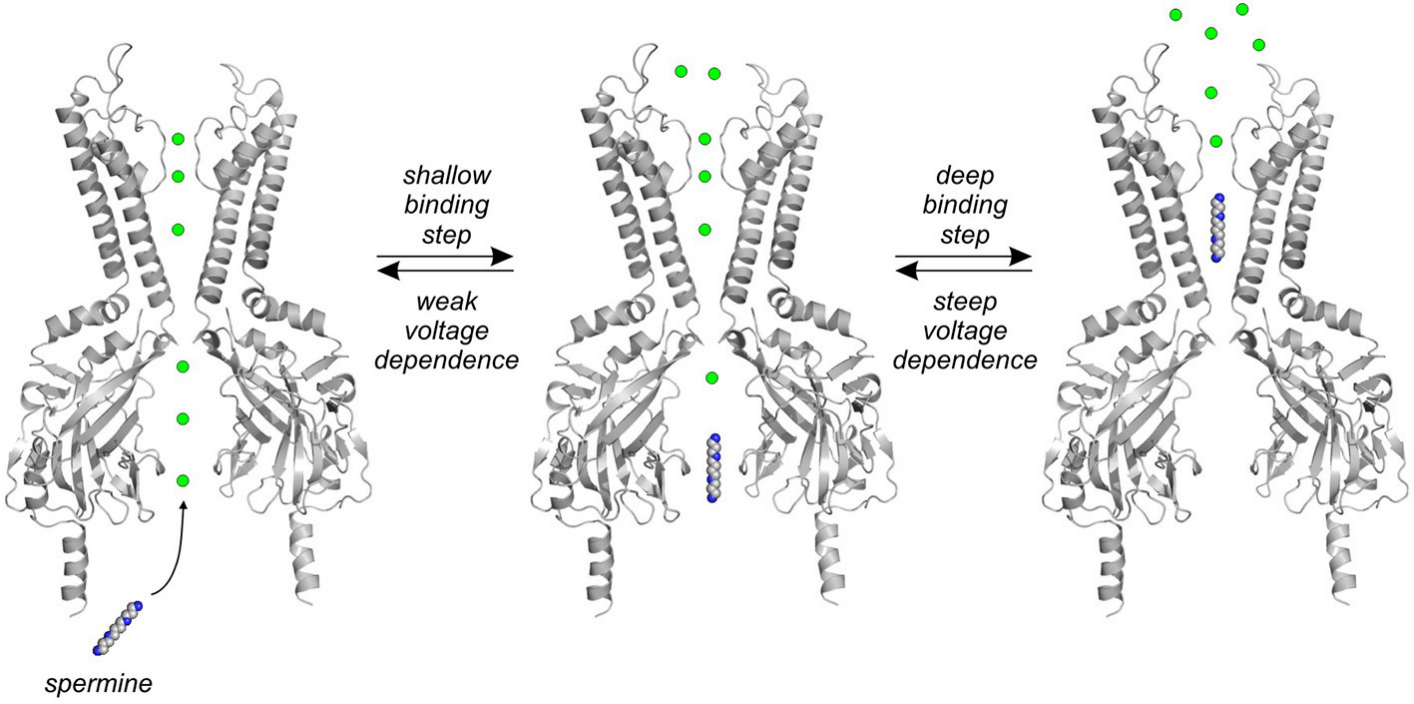
**Schematic diagram of polyamine migration and coupled ion movement**. Spermine and other polyamines migrate through the channel pore toward a binding site in the TMD, and displace occupant ions ahead of them in the pore. The first binding step involves a low affinity interaction between spermine and various residues in the CTD. The second “deep” binding step involves polyamine migration from the cytoplasmic pore into the channel inner cavity, with a steeper voltage dependence (likely because of displacement of a greater number of ions—note that the specific arrangement of ions relative to the blocker is unknown).

Observations such as these have led to the hypothesis that voltage dependence of polyamine block arises primarily from displacement of potassium ions by the migrating polyamine as it approaches its terminal binding site deep within the Kir pore (shown schematically in Figure 3—note that the exact locations of permeant ions, and their arrangement relative to migrating blockers, are not known). That is, as the blocker moves through the channel pore, obligate displacement of permeant ions ahead of the blocker results in charge movement through the membrane field (even though the blocker itself has traversed little or no fraction of the field). This coupled movement of polyamines (or other blockers) and permeating potassium ions in the pore is consistent with classic observations that polyamine block is tightly coupled to the potassium reversal potential, such that increasing the extracellular potassium concentration shifts the onset of polyamine block to more positive membrane voltages (Hagiwara et al., 1976; Leech and Stanfield, 1981; Lopatin and Nichols, 1996). It is noteworthy that the effective valence of block of Kir2.1 channels falls from ~4 to 5 elementary charges for long blockers, to ~2 charges for shorter blockers like putrescine (4 carbons). Various explanations have been proposed to account for this reduction of effective valence of block. One possibility is that the shorter blockers do not migrate as deeply into the channel pore as longer blockers, and thereby displace fewer ions as they reach their binding site (Pearson and Nichols, 1998; Guo et al., 2003). Another proposal has been that the shorter blockers may not efficiently occlude the channel pore, particularly at shallow sites, and thereby some “slippage” or “bypass” of permeating ions may contribute to the smaller observed effective valence (Shin et al., 2005; Xu et al., 2009).

Additional important insights have arisen from the characterization of extended polyamine analogs (with >4 amine groups, Figure 1D) and analogs with altered terminal amines (Figure 1C). Importantly, despite their considerably higher charge, extended polyamine analogs such as the deca-amines depicted in Figure 1D do not exhibit steeper voltage-dependence than spermine (Loussouarn et al., 2005; Kurata et al., 2006; Li et al., 2013). This is also consistent with the notion that the effective valence associated with polyamine block does not depend on movement of the entire blocker through the transmembrane field, but rather the displacement of coupled permeating ions as the blocker migrates through the channel pore. Lastly, the effects of modification of the terminal amines of spermine or other polyamine analogs have been tested (Figure 1C), typically resulting in weaker blocker affinity for the channel, while maintaining a steep voltage dependence of block (Shin and Lu, 2005; Kurata et al., 2008).

## Kinetic models of polyamine block, and their physical interpretation

### Kinetic models of steeply voltage dependent polyamine block

Kinetic models that reproduce the voltage and concentration dependence of polyamine block of Kir2.1 channels require at least two different equilibria, generally interpreted to indicate two or more distinct polyamine binding sites (Lopatin et al., 1995). In most published kinetic models these binding events are arranged sequentially: a superficial binding step in the CTD occurs first, followed by a second displacement of the blocker from the shallow binding site to a deeper and more stable binding site in the vicinity of the rectification controller in the TMD (Lopatin et al., 1995; Shin and Lu, 2005; Kurata et al., 2007) (shown schematically in Figure 3). In conductance-voltage relationships describing spermine block, these multiple binding sites are manifested as a shallow (weakly voltage dependent) component of block that is apparent at negative voltages in high polyamine concentrations, and a steeper (strongly voltage dependent) component that reflects polyamine binding to the high affinity TMD site. However, it should be noted that these features of spermine block have also been interpreted with alternative models invoking distinct populations of channels with high and low spermine affinities (Yan and Ishihara, 2005).

In kinetic models of spermine binding with multiple (sequential) binding sites, the first blocking step has very weak voltage dependence, with an effective valence typically less than 1, indicating the movement of 1 or less ions through the membrane field ahead of the blocker (Figure 3). Furthermore, interactions between spermine and this shallow binding site are of very low affinity. It is generally agreed that the first shallow blocking step involves interactions between polyamines and numerous residues in the CTD (Section Regulation of polyamine block by residues in the cytoplasmic domain). This shallow binding step can be specifically abolished by mutations of F254 or D255 in the “lower ring” of the CTD of Kir2.1, while mutations in the “upper ring” of acidic charges (Figure 2D) have mixed effects on spermine binding in the TMD and CTD sites, along with effects on ion permeation (Taglialatela et al., 1995; Guo and Lu, 2003; Shin et al., 2005; Kurata et al., 2007). The weak affinity of polyamine binding in the CTD suggests that polyamine interactions with this channel region are very brief, with some studies suggesting that these interactions generate incomplete blocked states (Xie et al., 2003). However, some structural approaches have begun to address the fine details of spermine binding in the CTD. A NMR study of spermine binding to the isolated Kir3.1 CTD has suggested that spermine binding near D260 (Kir2.1 residue D259) triggers a conformational rearrangement of the CTD (Osawa et al., 2009). In addition, one study has reported crystallization of the KirBac3.1 channel, in the presence of a high concentration of spermine (50 mM), with density ascribed to spermine in the CTD (Clarke et al., 2010). However, KirBac3.1 lacks several important spermine interacting residues in this region, including the entire “lower ring” cluster, and the equivalent of Kir2.1 residue E224. Moreover, KirBac3.1 has no reported spermine sensitivity, and the fairly closely related KirBac1.1 is insensitive to spermine (Cheng et al., 2009).

Most of the voltage dependence associated with polyamine block arises from a second binding step, involving the migration of the blocker from the shallow site in the CTD toward the inner cavity region of the TMD. This movement is likely accompanied by displacement of numerous permeant ions (Figure 3) (Pearson and Nichols, 1998; Guo et al., 2003). The only reported crystallographic evidence of a spermine binding site in the inner cavity is again in KirBac3.1 (Clarke et al., 2010), but as mentioned, these channels are likely highly insensitive to spermine and so it is unclear how this structural data relates to spermine block of strong inward rectifiers like Kir2.1. Moreover, polyamine binding is extremely sensitive to voltage and permeant ion concentrations, and these effects will need to be carefully considered when interpreting structural data that may emerge in the future (Lopatin and Nichols, 1996). In any case, until more detailed structural insights into spermine binding become available, most have settled for trying to interpret a variety of functional data to constrain a description of the spermine binding site.

### Divergent models of spermine binding in the inner cavity site

The details of polyamine binding in the inner cavity, in the vicinity of the rectification controller residue, has been among the most extensively debated questions in studies of polyamine block of Kir channels. Given the varied approaches taken to solve this problem, it is not surprising that a variety of structural models of polyamine binding have been proposed. We hope to summarize these ideas and offer suggestions for future development of studies on this mechanism.

**Figure 4:**
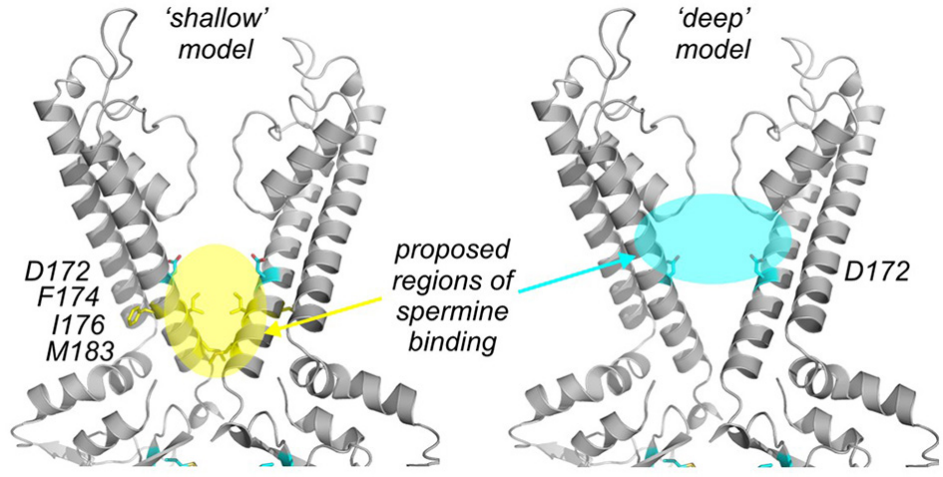
**Contrasting models of the deep spermine binding site in Kir2.1. (left panel)** The “shalllow model” proposes a binding region “below” the rectification controller position, highlighted in yellow, along with residues in this region that have been demonstrated to reduce polyamine blocker affinity. **(right panel)** The “deep” model proposes a binding site highlighted in cyan, supported by experiments testing the interactions between polyamine block and modification of substituted pore-lining cysteines.

One extensive set of studies has proposed a polyamine binding site with the leading end of the blocker oriented in the vicinity of the “rectification controller” position, and the trailing end of the blocker located close to residue M183 (Guo and Lu, 2003; Shin and Lu, 2005) (we refer to this as the “shallow” model, Figure 4). Early work in support of this hypothesis systematically characterized the energetics of block by bis-amine compounds of different lengths, in multiple Kir2.1 mutants, and demonstrated a sharply resolved optimal energetic coupling of 1,9-diaminononane with the D172 and E224/E299 residues, leading to the suggestion that the blocker spans the distance between the “rectification controller” and “upper ring” clusters of acidic residues (Guo and Lu, 2003). This explanation is difficult to reconcile with the structures of full-length Kir channels that have since emerged (Hansen et al., 2011), because the distance between the D172 and E224/E299 residues is much longer than predicted by this functional data, and alternative explanations for this strong energetic coupling were also proposed (John et al., 2004). In other studies supporting a shallow binding model, an alanine scan of the M2 helix flanking the pore highlighted four mutations that reduce spermine affinity (D172A, with weaker effects arising from F174A, I176A, and M183A, highlighted in Figure 4, yellow), all located in the lower half of the inner cavity. Extension of this alanine-scanning approach led to the demonstration that compound mutation of five residues (D172, I176, M183, F254, and E299) spanning the CTD and TMD eliminates spermine sensitivity of Kir2.1. The proposed interpretation of these data was that residue D172, along with positions located closer to the cytoplasmic entrance (M183, E224, E299), flanked spermine in the inner cavity binding site (Xu et al., 2009), primarily because these mutagenic scanning approaches did not reveal any residues deeper in the pore that significantly affect polyamine block. A final motivation for the shallow binding hypothesis is that polyamine analogs with sterically expanded termini (e.g., decamethonium, or “bis-QA-C10,” Figure 1C) can recapitulate the steep voltage dependence of block observed with spermine. These expanded termini should preclude entry into the selectivity filter, therefore the data indicate that blocker migration into selectivity filter is not needed in order to generate steeply voltage dependent block (Shin and Lu, 2005).

**Figure 5:**
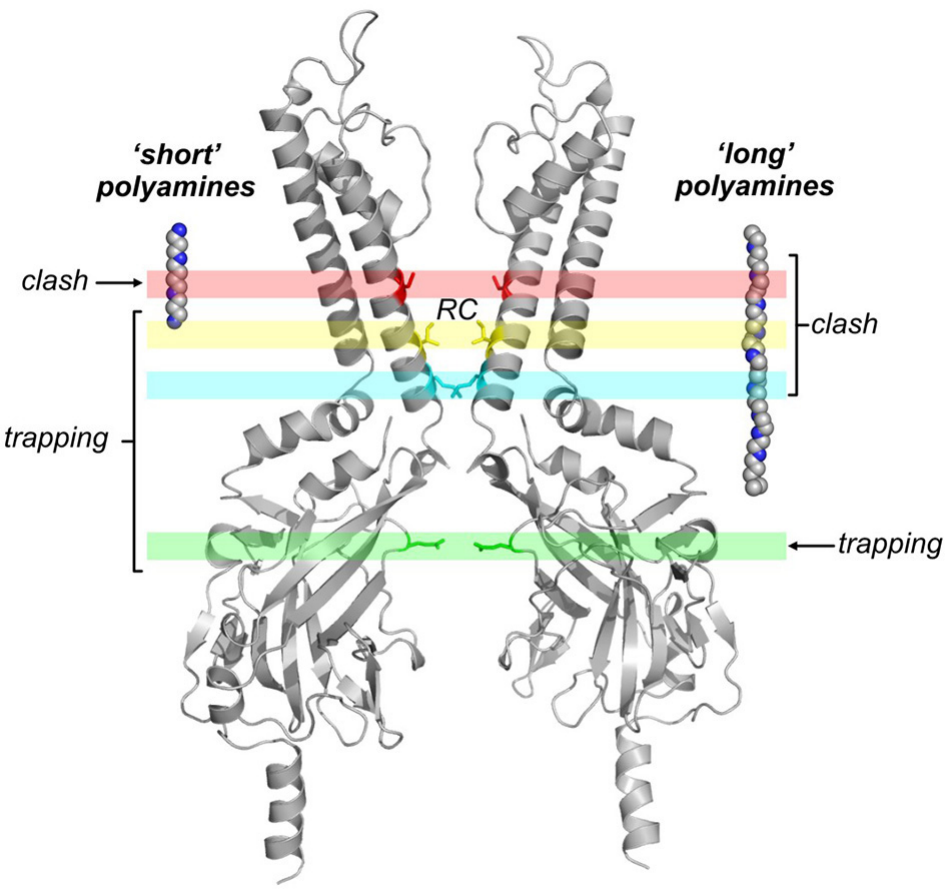
**Blocker trapping approaches highlight a deep polyamine binding site**. This image summarizes the effects of MTS modification of pore-lining cysteines on kinetics of polyamine binding and unbinding, highlighted by two recent studies (Kurata et al., 2010, 2013). At positions deep in the pore (red band), the potency of all tested blockers is significantly reduced after modification with positively charged MTS reagents (“clash”). At intermediate positions (blue and yellow bands), short polyamines could be “trapped” in the inner cavity by modification just below the rectification controller position (inner cavity modifier), while blockade by long polyamine analogs is dramatically disrupted because the charged modifying reagent clashes with longer blockers. At the most superficial modification position tested (green band), both the long and short polyamines can be trapped. “RC” indicates the pore depth of the “rectification controller” position.

Several other groups, including our own, have suggested a somewhat different orientation of spermine in the inner cavity, largely based on the effects of chemical modification of introduced cysteine residues at pore-lining positions (Figure 4, cyan) (Chang et al., 2003; John et al., 2004; Kurata et al., 2004, 2006, 2008, 2010, 2013). Our most recent work applying these methods has demonstrated that introduction of positively charged MTS adducts on the intracellular side of the inner cavity (“below” the rectification controller residue) can markedly decelerate spermine unbinding in both Kir2.1 and Kir6.2[N160D] channels. We have interpreted this observation as a “trapping” effect of the blocker in a deep binding site (Kurata et al., 2010, 2013), in which introduction of positively charged adducts in a suitable position can introduce an energetic barrier that impedes blocker entry to, or exit from the binding site. These observations suggest that spermine migrates to a position deep in the inner cavity, between the rectification controller and selectivity filter. Reinforcing this view, the phenomenon of blocker “trapping” by MTS modification correlates blockers of different lengths with modification at different pore depths. Specifically, modification of positions deep in the pore (red band in Figure 5) overlaps and “clashes” with the blocker binding site, leading to a reduction of blocker affinity for both long and short polyamines (Kurata et al., 2010, 2013). At certain intermediate positions (i.e., yellow and blue bands, Figure 5), shorter blockers like spermine are “trapped” by modification (exhibit slow binding/unbinding kinetics), whereas longer blockers (such as the deca-amines in Figure 1D) continue to “clash” with the charged adduct. At the most shallow modification position tested (in the CTD of Kir6.2[N160D] channels green band, Figure 5), both short and long polyamines can be “trapped” by introduction of a charged adduct (Kurata et al., 2013). Similar approaches have been applied in both Kir2.1 and Kir6.2[N160D] channels, with the striking consistent finding that spermine can be “trapped” by MTSET modification of a cysteine substituted one helical turn below the “rectification controller” (L164C in Kir6.2, I176C in Kir2.1) (Kurata et al., 2010, 2013). Other data in support of a stable binding site deep in the inner cavity is that polyamine blockers can inhibit cysteine modification by MTS reagents at deep sites in the Kir pore (a phenomenon referred to as “blocker protection”) (del Camino et al., 2000), but not at positions closer to the cytoplasmic entrance. Consistent with this, longer polyamine analogs are also able to “protect” cysteines substituted at positions closer to the cytoplasmic pore entrance (Chang et al., 2003; Kurata et al., 2006, 2008). Overall, these experimental findings are consistent with polyamine blockers primarily occupying space between the “rectification controller” position and the selectivity filter.

**Figure 6:**
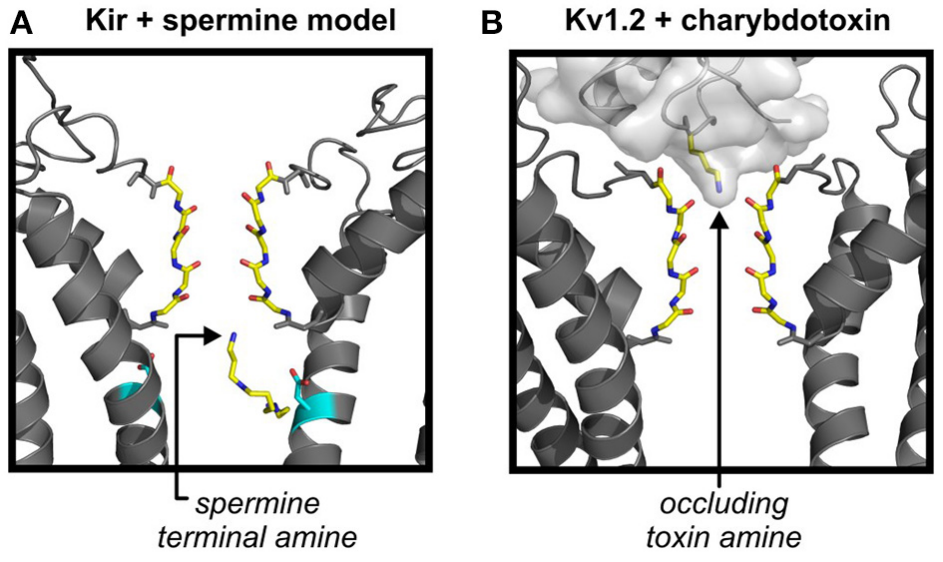
**Does charybdotoxin binding reveal details of amine interactions with the selectivity filter? (A)** Depiction of a previously published molecular model of Kir2.1 with a polyamine bound in a deep site in the Kir inner cavity (Kurata et al., 2008). The trailing end of the polyamine is anchored around the “rectification controller” position, while the leading end engages with the most superficial intracellular aspect of the selectivity filter. Docking simulations were carried out using Autodock, and multiple molecular models of Kir2.1 (depicted) or Kir6.2[N160D], based on the KirBac1.1 crystal structure. **(B)** Recent crystal structure of charybdotoxin in complex with Kv1.2, illustrating an essential lysine interacting with the most superficial extracellular aspect of the selectivity filter. Both spermine and the occluding lysine side chain have similar functional groups (protonatable amines) at their termini, and both blocker types are particularly sensitive to ion concentrations on the “trans” side.

A satisfactory physical description of the spermine binding site should describe the physical orientation of spermine within the channel, and account for the steep voltage dependence of spermine block. In either of the above hypotheses describing the deep spermine binding site, two important points are worth considering as studies move forward. Firstly, spermine and other polyamines are very flexible compounds with numerous torsionable bonds that can sample a variety of conformations—there may not be a single defined “site” or orientation of spermine bound in the TMD. In this regard, one criticism of the interpretation of a deep binding site (between the “rectification controller” and selectivity filter, Figure 4, cyan) has been that a fully extended spermine blocker is considerably longer than the distance between these two sites (Shin and Lu, 2005). However, a few modest bond rotations enable spermine to readily occupy this region deep in the channel (Figure 6A) (Kurata et al., 2008)—this more realistic treatment of the conformational space sampled by spermine may help to reconcile the contrasting viewpoints described above. Secondly, the primary objective of generating structural models of polyamine block is to understand the molecular basis for the steep voltage-dependence of the process—this is inherently tied to the location of permeating ions in the pore, and the mechanisms that couple ion and blocker displacement. These details of polyamine block and ion interactions are not well accounted for in prevailing models, and will continue to be investigated.

## Channelopathies and physiological roles of polyamine sensitive Kir channels

The following sections are intended to provide some brief physiological context to the biophysical aspects of polyamine block discussed thus far. Early characterization of inward rectifiers primarily involved studies in cardiac and skeletal muscle, and played a prominent role in the evolution of Hodgkin-Huxley models to describe more complex action potentials in tissues like cardiac muscle (Noble, 1962a,b). Consequently, considerable attention continues to be devoted to the role of Kir channels in cardiac function (Nichols et al., 1996; Dhamoon and Jalife, 2005). However, currents generated by Kir channels have been described in other excitable cell types, and contribute to electrical activity or ion transport in many specialized cells including neurons, glia, and vascular smooth muscle (Hibino et al., 2010). Though not meant to be exhaustive, the following sections provide a perspective on some channelopathies and physiological roles of strongly rectifying Kir channels.

### Cardiac arrhythmias related to Kir2.x channels and polyamine block

Counteracting effects of depolarizing (Ca^2+^, Na^+^) and hyperpolarizing (K^+^) currents are responsible for shaping the “plateau” phase of the cardiac action potential and repolarization to a stable resting membrane potential (Nerbonne and Kass, 2005). Strongly rectifying channels composed of Kir2.x subunits (often in heteromeric combinations) underlie a strongly rectifying conductance, typically referred to as IK1 in cardiac myocytes, which contributes to repolarization during late phases of the action potential and maintains the resting membrane potential during diastole (Zaritsky et al., 2001; McLerie and Lopatin, 2003; Zobel et al., 2003). This period of rest during the cardiac excitation cycle is important to allow recovery of voltage gated sodium channels from inactivation and trigger subsequent heartbeats (Deo et al., 2013). The importance of the IK1 current for normal cardiac function is perhaps best illustrated by the bidirectional effects on cardiac action potential duration arising from either loss-of-function (Plaster et al., 2001) or gain-of-function (Priori et al., 2005) mutations in Kir2.1. A recently recognized channelopathy that is directly related to polyamine block is the short QT syndrome arising from mutations of Kir2.1 that cause a gain-of-function phenotype by disrupting polyamine block. Such mutations have been identified in several pedigrees exhibiting inherited short QT syndrome and this underlying cause of electrical disruption is categorized as SQT3 (Priori et al., 2005; Patel and Pavri, 2009; Hattori et al., 2012; Deo et al., 2013). In these scenarios, the Kir2.1 mutation weakens polyamine block, leading to an increased repolarizing influence and early termination of the cardiac action potential.

Only three mutations have been identified to date in SQT3 patients. The first reported mutation caused charge neutralization of the “rectification controller” residue (D172N), whose functional role was described in Section Regulation of polyamine block by residues in the transmembrane domain (Priori et al., 2005). Two recent studies have highlighted mutations (E299V, M301K) near the G-loop and “upper ring” of the CTD (highlighted in Figure 2D) (Hattori et al., 2012; Deo et al., 2013). We presume that other mutations that control affinity and kinetics of polyamine block could also generate a cardiac phenotype, and with growing recognition of the genetic basis for short QT and associated cardiac arrhythmias, more causative mutations may be reported and characterized. A genetic counterpoint to the short QT syndrome is the large number of loss-of-function mutations of Kir2.1 that have been identified in patients with Andersen-Tawil syndrome. These patients exhibit a long QT phenotype classified as LQT7 (Tristani-Firouzi et al., 2002; Decher et al., 2007), consistent with the predicted effects of loss of a repolarizing influence on cardiac action potential duration. Taken together, these outcomes demonstrate the role of strongly rectifying Kir2.x channels in cardiac muscle, as tuning the activity of these channels can either prolong or shorten the cardiac action potential.

The role of Kir2.x channels in action potential repolarization also highlights the possibility for tuning of cardiac function by post-translational modification of these channels. This may sometimes be linked to certain pathological states, as revealed by the recent studies of nitrosylation of Kir2.1 (at residue cysteine 76). This NO dependent post-translational modification upregulates heterologous Kir2.1 currents and endogenous IK1 from isolated myocytes, and is reduced in tissue from patients suffering from chronic atrial fibrillation (Gomez et al., 2009). Other signaling mechanisms that influence Kir2.x channels dictate the cardiac IK1 response to adrenergic stimulation, which likely involves integration of PKA and PKC signaling cascades in heteromeric Kir2.x channels. For example, adrenergic stimulation tends to reduce IK1 currents overall (Koumi et al., 1995; Sosunov et al., 2004), although different Kir2.x subunits have varied responses to PKC and PKA (Henry et al., 1996; Karle et al., 2002; Zitron et al., 2004, 2008; Scherer et al., 2007). Other signaling cascades that alter PIP2 levels (e.g., via phospholipase C activation) will have general effects on all Kir channel subunit types (Rohacs et al., 1999). Lastly, an important emerging aspect of Kir channel regulation of cardiac function has been the recognition that Kir2.x channels (and other Kirs) are unintended targets of certain drugs such as the anti-malarial chloroquine, and their inhibition may lead to development of ventricular arrhythmias (Rodriguez-Menchaca et al., 2008). However, blockade of IK1 and other Kir mediated currents by chloroquine and related compounds has also been suggested as a potential anti-arrhythmic strategy in certain conditions (Noujaim et al., 2010). Overall, altered cardiac IK1 current density or function can arise by genetic mutations, moment-to-moment regulation, and pharmacological modulation, with consequences on cardiac action potential duration and arrhythmogenesis.

### Strongly rectifying channels in development of bone and muscle

Significant perturbative developmental effects arise from loss-of-function mutations in the strongly rectifying Kir2.1 channel. These are interesting to consider because they have not yet been linked in an obvious causative way to the moment-to-moment electrical function of these channels. These effects are particularly apparent in the development of bone and muscle. Carriers of loss-of-function Kir2.1 mutations (Andersen-Tawil syndrome) typically exhibit morphological abnormalities including short stature, facial abnormalities, and unusual bone structure in their extremities (Plaster et al., 2001; Tristani-Firouzi and Etheridge, 2010). Moreover, genetic deletion of Kir2.1 (but not Kir2.2) in mice causes a cleft palate phenotype that leads to death of pups shortly after birth (Zaritsky et al., 2000). The specific role of Kir2.1 channels in bone development is not yet well understood, although it has been suggested that the loss of a K^+^ conductance may influence H^+^ transport in osteoclasts, thereby influencing bone remodeling processes (Hibino et al., 2010). Overall, these clinical findings suggest that strongly rectifying Kir channels have significant effects in development of tissues (such as bone) that are not generally considered to be electrically excitable.

Some of the fine details of Kir2.1 regulation in development have been documented in very early stages of muscle differentiation. Specifically, Kir2.1 channels are inhibited by phosphorylation of tyrosine 242 in early stages of muscle development (Wischmeyer et al., 1998; Fischer-Lougheed et al., 2001). Subsequent dephosphorylation of this tyrosine activates the channels leading to membrane hyperpolarization, thereby generating a strong driving force for influx of Ca^2+^ ions (through a small fraction of voltage-gated calcium channels that remain open at this membrane potential). This Ca^2+^ influx contributes to an early signal/trigger for the differentiation of myoblasts (Konig et al., 2004; Hinard et al., 2008). There have been diverging reports, however, as to whether phosphorylation of Tyr242 impacts channel function by effects on gating or trafficking (Tong et al., 2001). Specific problems with muscle development have not been well described in Andersen's syndrome, although these patients often exhibit episodes of muscle weakness/paralysis (Plaster et al., 2001). It remains unclear whether similar principles associated with muscle differentiation are also implicated in the bone development phenotype of Andersen-Tawil syndrome. Nevertheless, the apparent roles of Kir channels in development of bone and muscle highlight the importance of understanding ion channel function in contexts beyond rapid electrical signaling.

### Regulation of Kir channel function by altered polyamine metabolism

Another potential underlying cause of altered Kir channel function is disruption of endogenous polyamine metabolism, which might arise via genetic mutations of polyamine biosynthetic enzymes, or pharmacological inhibition. Pharmacological modulation of endogenous polyamine levels (using ornithine decarboxylase inhibitors) can significantly alter the kinetics and magnitude of current from heterologously expressed Kir2.1 and Kir2.3 channels (Shyng et al., 1996). More importantly, physiological consequences of altered polyamine levels are apparent in animal models and human patients exhibiting defective polyamine metabolism. Numerous defects arise in the “gyro” mouse model (carrying a deletion of an X chromosome region containing the spermine synthase gene, and a second gene involved in phosphate metabolism), including poor physical development, neurological defects, sterility, and shortened lifespan. Remarkably, many of these traits can be rescued by transgenic overexpression of spermine synthase, illustrating the widespread functional importance of appropriate polyamine biosynthesis (Wang et al., 2004). A rare but dramatic correlate in humans is the X-linked disease Snyder-Robinson-Syndrome (SRS), caused by partial or complete loss-of-function of the spermine synthase gene, leading to severe mental retardation, poor muscle development and hypotonia, and bone defects (Cason et al., 2003). As mentioned above for developmental defects in Andersen's syndrome, it is unclear how or whether strongly-rectifying ion channels are involved in generating the developmental defects associated with SRS (and it must be noted that polyamines have numerous physiological roles in addition to blockade of Kir channels). Nevertheless, it is interesting that SRS and Andersen's syndrome have some overlap in terms of the organ systems affected (Schwartz et al., 2011; Peron et al., 2013), suggesting that the roles of polyamine sensitive Kir channels should continue to be investigated in the context of tissue differentiation and development. Interestingly, while a polyamine deficiency might be expected to mimic gain-of-function mutations of Kir channels, there are no reports for a “short-QT” phenotype in SRS-affected individuals. This might arise because the spermine synthase deficiency causes a marked elevation of the spermidine:spermine ratio (Sowell et al., 2011). Spermidine is an effective blocker of Kir channels (slightly less effective than spermine), thus the elevated spermidine levels arising in spermine synthase deficient organisms may compensate for a lack of spermine in terms of Kir channel block (Lopatin et al., 2000).

## What is left to learn about polyamine block of Kir channels?

Structure-function work based on site directed mutagenesis and electrophysiological studies has led to a concrete description of the amino acids involved in controlling kinetics and affinity of polyamine block. However, numerous unanswered questions linger regarding the specific chemical forces that dictate polyamine block, and the exact nature of interactions between polyamines and acidic amino acid side chains that influence polyamine block. To conclude this review, we have highlighted what we perceive to be pertinent lingering questions related to the mechanism of polyamine block of Kir channels.

### What is the pKa of the “rectification controller” and other functionally important pore-lining acidic residues?

Recent studies in nicotinic acetylcholine receptors and other model systems have highlighted the context-dependence of the pKa of glutamate and aspartate side chains (Cymes and Grosman, 2011). When forced into close proximity, the pKa of carboxylates may shift by several pH units, permitting them to be predominantly uncharged at physiological pH (Lindman et al., 2006, 2007). In the Kir channel pore, many rings of “charge” lie at narrow pore-lining apertures (Figures 2C,D), and while it is generally assumed that they are negatively charged, little has been done to explicitly measure their protonation state or its influence on spermine block. Recent work in nicotinic receptors highlights that this assumption may be premature (Cymes and Grosman, 2008, 2012). Similarly, introduction of acidic residues at certain pore-lining positions of the Kir6.2 channel have strongly indicated a marked pKa shift of the carboxylate side chain (Khurana et al., 2011). In part, the paucity of information on the protonation of pore-lining residues in Kir channels may be due to the restricted chemical tools available to answer these questions. For instance, conservative substitutions of Asp or Glu to Asn or Gln remove the potential to carry a formal charge, but also significantly alter the hydrogen bonding properties of the side chain (Pless et al., 2011), and this may be an important determinant of polyamine affinity (Kurata et al., 2010). Of particular relevance to polyamine block, the polarized N-H bonds of the amide functional group (of substituted Asn side chains in the widely characterized Kir2.1[D172N] mutant) may clash significantly with N-H bonds of polyamines (Pless et al., 2011). Recent application of unnatural amino acid mutagenesis in K^+^ channels might address these questions by enabling substitution of more subtle derivatives of carboxylate side chains, such as the uncharged glutamate isostere nitrohomoalanine (which replaces the carboxylate with a uncharged non-protonatable nitro group) (Pless et al., 2011). Investigation of this question will provide new insights into the chemical mechanism of polyamine interactions with Kir channels.

### Do blocker amines interact with the selectivity filter? if so, how?

This is an interesting question that has been debated significantly in the context of the stable “deep” spermine binding site (Section Divergent models of spermine binding in the inner cavity site), and may be cast in a different light by recent crystal structures of Kv1.2 in complex with charybdotoxin (Banerjee et al., 2013). Many previous studies inferred close proximity of polyamines and the selectivity filter based on various mutagenic approaches and cysteine modification methods—but these could not explicitly test the question of whether spermine significantly migrates into the selectivity filter (Chang et al., 2003; Kurata et al., 2004, 2010). It is also true that polyamine analogs with “bulky” ends mimic the voltage dependence of spermine block (Shin and Lu, 2005), as do polyamine analogs with fewer charges (e.g., 1,10-diaminodecane) (Pearson and Nichols, 1998), indicating that movement of the charged polyamine blocker through the selectivity filter cannot account for the bulk of the voltage-dependence of block (Guo and Lu, 2003; Lu, 2004).

Recent structures of charybdotoxin bound to Kv1.2 illustrate that the terminal amine of a critical side chain lysine is coordinated by the external K^+^ binding site of the Kv1.2 selectivity filter (Figure 6B), and alters the distribution of ions in the filter (Banerjee et al., 2013). This interaction between the toxin and ions in the filter has been proposed to underlie the pronounced effects of intracellular K^+^ concentration (“trans” to the toxin site) on toxin affinity. Although polyamines block Kir channels from the intracellular side, the same principles may apply, as polyamines exhibit a pronounced dependence on K^+^ concentration on the *trans* (i.e., extracellular) side (Lopatin and Nichols, 1996). A similar blocker-dependent redistribution of K^+^ ions in the selectivity filter has been proposed to account for the coupling between external K^+^ concentration and internal Mg^2+^ block of Kir1.1 channels (Yang et al., 2012). Thus, the recent structure of the occluding amine of charybdotoxin may provide some clues as to the arrangement of spermine or other polyamines in their ultimate blocked state, with an amine superficially engaged with a K^+^ binding site on the intracellular side of the selectivity filter (without necessarily entering deep into the selectivity filter). Such an arrangement was suggested in earlier studies aimed at mapping inner cavity residues that are protected against MTS reagent modification by spermine occupancy (Kurata et al., 2008). The hypothesis emerging from this work was that spermine may be anchored by an interaction between one or two amines and the “rectification controller” in the inner cavity, with other amines engaging peripherally with the selectivity filter (Figure 6A). A final point to reiterate about these blocked conformations of spermine is that the blocker is very flexible, with many torsionable bonds, and need not be restricted to the fully extended linear conformation that is often used while interpreting structure-function experiments involving polyamines (Figure 6A).

## Summary

Polyamine blockade is a well-recognized mechanism able to generate steep voltage-dependence of ion channels in excitable tissues. Mutations that disrupt the function of strongly rectifying channels have pronounced effects on the electrical properties of excitable cells, and on aspects of tissue development and growth. The molecular details of polyamine block have been dissected with conventional structure-function approaches, and we anticipate that application of new approaches and emerging methods will add important details to current models of this process.

### Conflict of interest statement

The authors declare that the research was conducted in the absence of any commercial or financial relationships that could be construed as a potential conflict of interest.
